# Oligodontia Management in a Resource-Limited Setting: Two Case Reports and Review of Literature

**DOI:** 10.1155/crid/5519222

**Published:** 2025-09-16

**Authors:** A. B. Malami, B. E. Ogbozor, C. C. Okolo, A. O. Aborisade, U. B. Mahmud, A. A. Abulfathi, Y. I. Adeyemo

**Affiliations:** ^1^Department of Child Dental Health, Bayero University, Kano, Kano State, Nigeria; ^2^Department of Child Dental Health, Aminu Kano Teaching Hospital, Kano, Kano State, Nigeria; ^3^Department of Oral and Maxillofacial Surgery, University of Nigeria–Enugu Campus, Enugu, Enugu State, Nigeria; ^4^Department of Oral and Maxillofacial Surgery, University of Nigeria Teaching Hospital, Enugu, Enugu State, Nigeria; ^5^Department of Oral Diagnostic Sciences, Bayero University, Kano, Kano State, Nigeria; ^6^Department of Oral Diagnostic Sciences, Aminu Kano Teaching Hospital, Kano, Kano State, Nigeria

**Keywords:** developmental abnormality, hypodontia, oligodontia, resource-limited setting, teeth agenesis

## Abstract

**Introduction:** Oligodontia represents the developmental absence of six or more teeth, posing significant challenges for masticatory function, speech, and psychosocial well-being. While extensively documented in developed countries, limited reports exist from resource-constrained settings in Africa. This study presents two pediatric cases of oligodontia managed with available resources and analyzes their clinical presentations against current literature. This case report was prepared following the CARE guidelines to ensure methodological rigor and completeness.

**Cases and Interventions:** Case 1, a 10-year-old female with a history of missing anterior teeth from birth, with no associated systemic abnormalities and no contributory family history, but with deranged alkaline phosphatase. To address masticatory function, speech, and esthetic problems, removable partial dentures were fabricated for both jaws. Case 2, a 10-year-old female with a family history of congenitally missing teeth but no other features of syndromic oligodontia, with an associated crown fracture involving the enamel, dentine, and pulp of the maxillary right central incisor, peg-shaped maxillary right lateral incisor, and retained mandibular central incisors. Systemic features of mild acanthosis nigricans, nail abnormalities, and hypohidrosis were observed with deranged alkaline phosphatase. To restore speech and masticatory function, root canal treatment and postretained crown restoration of the maxillary right central incisor and composite resin restoration for the peg-shaped maxillary right lateral incisors and retained lower central incisor teeth were done.

**Conclusion:** Early diagnosis and multidisciplinary management of oligodontia significantly improve functional outcomes and quality of life. Resource limitations necessitated adaptive treatment approaches while maintaining therapeutic efficacy.

## 1. Introduction

Oligodontia, defined as the congenital absence of six or more permanent teeth excluding third molars, represents one of the most severe forms of tooth agenesis. This rare developmental anomaly affects approximately 0.084%–0.16% of the global population, with significant variations across geographic regions and ethnicities [1, 2]. The condition substantially impacts masticatory function, speech development, facial aesthetics, and the psychological well-being of affected individuals.

The etiopathogenesis of oligodontia involves complex interactions between environmental and genetic factors. The environmental factors such as hormonal influences, early childhood infections like rubella (German measles), trauma involving the alveolar processes of the jaws, irradiation to the maxillofacial region, tumors of the maxillofacial region, and thalidomide teratogen exposure in early pregnancy [3] may contribute to tooth agenesis. Oligodontia can arise either spontaneously or as an isolated condition linked to mutations in certain specific genes of the human genome [4]. The key genes including Paired Box 9 (PAX9), Latent TGF-*β* Binding Protein 3 (LTBP3), and Muscle Segment Homeobox 1 (MSX1) gene regulate craniofacial development, especially odontogenesis, through various signaling pathways [5–7]. Oligodontia has also been linked with syndromes demonstrating phenotypic features of rare conditions such as cleft lip/palate, orofacial digital syndrome, Down syndrome, ectodermal dysplasia, and Van der Woude syndrome [6, 8].

The diagnosis of oligodontia involves comprehensive clinical examinations, radiological investigations, and relevant blood chemistry assays. The features of syndromic and nonsyndromic oligodontia can be differentiated through a thorough physical assessment of nails, hair, eyes, oral mucosa, sweat glands, and probing for any congenital disorders, as none of these features will be seen in nonsyndromic oligodontia [9]. However, a baseline gene assay is also essential for definitive diagnosis. Radiological imaging findings on standard dental pantomogram, occlusal radiographs, cephalometry, and cone beam CT (CBCT) are very useful in this investigation. CBCT has the advantage of a three-dimensional representation of the maxillofacial region at a low dose of radiation exposure [10].

Management of oligodontia requires a multidisciplinary approach that integrates insights from pediatric dentistry, orthodontics, prosthodontics, geneticists, developmental biology, and pediatric medicine [11]. However, in resource-limited settings, treatment options are often constrained by economic factors, limited technology, and reduced access to specialized care. While oligodontia has been extensively studied in developed countries, documentation from African populations remains limited. This study presents two pediatric cases of oligodontia managed in a northern Nigerian tertiary center, analyzing their clinical presentations, treatment approaches, and outcomes within the context of current literature.

This study was approved by the Health Research Ethics Committee of Aminu Kano Teaching Hospital, Kano, Nigeria (Approval Number: NHREC/28/01/2020/AKTH/EC/3808) following the informed and written consent obtained from the patients' parents.

## 2. Case Reports

### 2.1. Clinical Case 1

#### 2.1.1. Patient's Information

A 10-year-old girl was referred to the dental center of our hospital from a general hospital in the remote local government district of Kano state, Nigeria. She presented to the pediatric dental clinic alongside her mother, with congenital absence of anterior teeth, associated with chewing difficulties and altered phonation. The patient exhibited low self-esteem due to aesthetic concerns. However, there was no history of trauma or previous extractions. There was no history of maternal exposure to radiation, consanguinity, or family history of tooth agenesis, and maternal pregnancy history was unremarkable. Perspiration was normal, ruling out hypo/hyperhidrosis-associated ectodermal dysplasia.

#### 2.1.2. Clinical Findings

General physical examination revealed normal hair distribution, nonbrittle nails, and well-hydrated skin, excluding ectodermal dysplasia.

**Figure 1:**
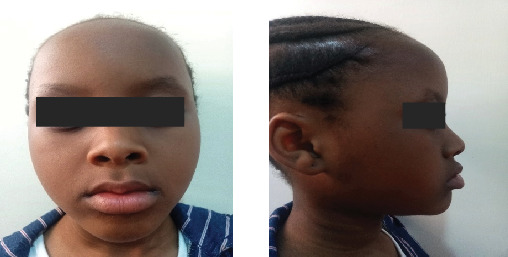
Clinical photograph of the subject showing her facial symmetry and facial profile.

Extra-oral examination revealed reduced lower facial height and a deep labio-mental fold, Type I skeletal relationship without facial asymmetry or observed lip abnormalities (Figure 1).

**Figure 2:**
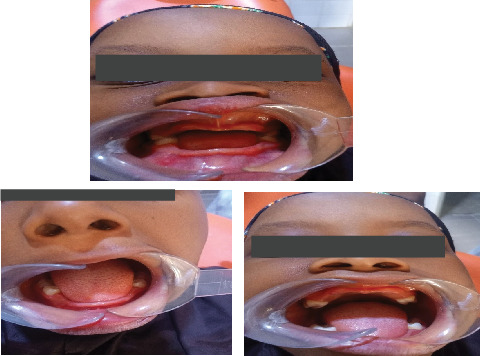
Clinical photographs of the subject showing congenital absence of incisors, canines, and premolars on the maxilla and mandible.

Intraoral assessment revealed reduced alveolar bone height, intact oral mucosa, complete hard and soft palates, and an anterior open bite. All primary second molars and first permanent molars were present and erupted (Figure 2).

#### 2.1.3. Diagnostic Assessment

**Figure 3:**
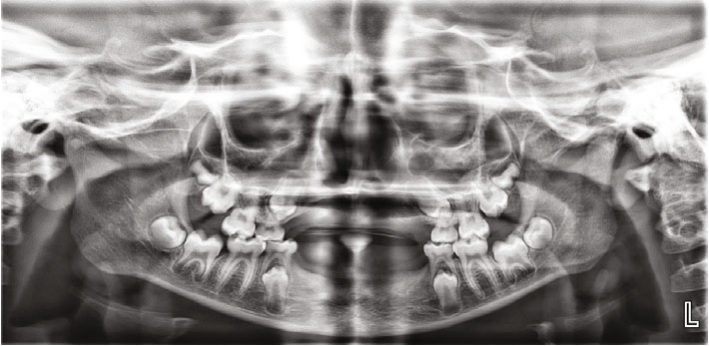
Dental pantomogram showing absence of permanent incisors, canines, and first premolars; erupted Teeth #55, #16, #65, #26, #75, #36, #85, and #46; and unerupted Teeth #15, #25, #35, #45, #17, #27, #37, and #47.

The dental pantomogram revealed the absence of permanent incisors, canines, and first premolars. Erupted teeth included Teeth #55, #16, #65, #26, #75, #36, #85, and #46, while Teeth #15, #25, #35, #45, #17, #27, #37, and #47 were unerupted, with all the tooth germs of second premolars positioned beneath the roots of the exfoliating second primary molars (Figure 3).

The patient was also referred to the ophthalmology clinic for a comprehensive eye examination, but the results were uneventful. Furthermore, serum biochemistry assay, including calcium, inorganic phosphate, alkaline phosphatase, and thyroid function tests, was within normal limits.

However, the limitation of resources in our center precluded advanced diagnostic techniques (‘Tooth agenesis panel'—next-generation sequencing [NGS]).

#### 2.1.4. Treatment

**Figure 4:**
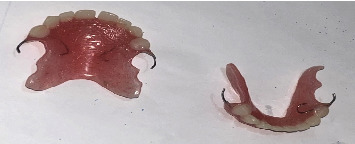
Upper and lower removable partial dentures.

**Figure 5:**
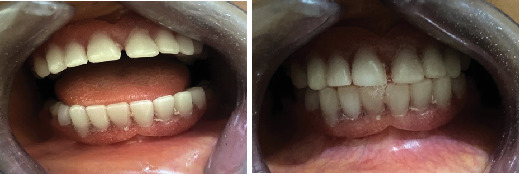
Upper and lower removable partial dentures in situ.

The treatment modality instituted for this patient included prosthetic replacement of the missing teeth with a removable partial denture for aesthetic and functional rehabilitation (Figures 4 and 5). A cast was made from an impression of the girl's oral tissues, from which the dentures were fabricated on their first visit. A few days later, the upper and lower partial dentures were delivered.

#### 2.1.5. Follow-Up and Outcomes

Regular weekly reviews with adequate prosthetic and psychological rehabilitation were instituted. The patient demonstrated improved speech clarity, enhanced chewing efficiency, and increased self-confidence posttreatment.

### 2.2. Clinical Case 2

#### 2.2.1. Patient's Information

A 10-year-old girl, accompanied by her parent, presented with spontaneous pain in a fractured upper anterior tooth following trauma 7 months prior. Additional concerns included small-sized anterior teeth and aesthetic dissatisfaction. There was no history of maternal exposure to radiation or consanguinity, but there was a positive family history of an elder sister with congenitally missing teeth, though not previously diagnosed with oligodontia.

#### 2.2.2. Clinical Findings

**Figure 6:**
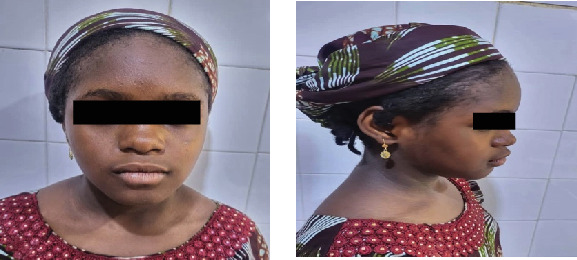
Clinical photographs of the patient showing her facial symmetry and facial profile.

**Figure 7:**
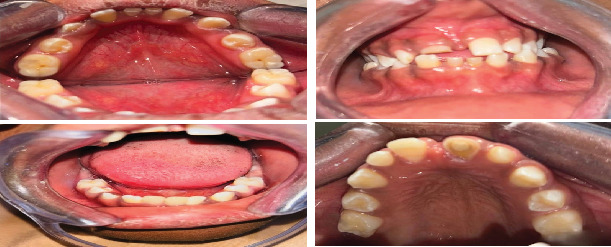
Clinical intraoral pictures showing intact soft and hard palates with peg-shaped Tooth #12, retained Teeth #71 and #81, and absent Teeth #36 and #46.

Physical examination revealed acanthosis nigricans, nail abnormalities, and hypohidrosis, suggesting possible ectodermal involvement. Extraoral examination revealed facial symmetry with normal skeletal relationships (Figure 6). Intraoral assessment demonstrated intact soft tissues, hard palate, crown fracture of Tooth #11 with pulpal involvement; peg-shaped tooth #12; retained primary Teeth #71 and #81; and absence of Teeth #26, #36, and #46 (Figure 7).

#### 2.2.3. Diagnostic Assessment

**Figure 8:**
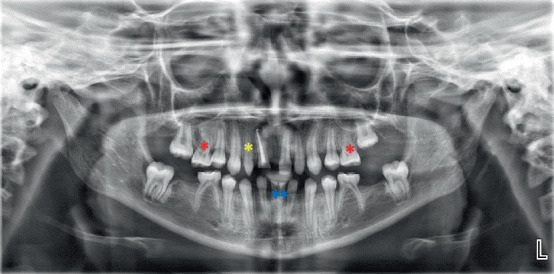
Dental pantomogram showing retained Teeth #71 and #81 (blue asterisk); absence of tooth germs for Teeth #15, #17, #25, #27, #31, #35, #36, #41, #45, and #46; Taurodont-like Teeth #16 and #26 (red asterisk); and peg-shaped Tooth #12 (yellow asterisk).

Dental pantomogram of the patient confirmed horizontal crown fracture of Tooth #11 with pulpal involvement and absence of teeth germ of the Teeth #15, #17, #25, #27, #31, #35, #36, #41, #45, and #46. It also showed retained Teeth #71 and #81; Teeth #55, #65, #75, and #85; and taurodont morphology of Teeth #16 and #26 (Figure 8). Furthermore, relevant laboratory blood chemistry assessments revealed only a slight derangement in alkaline phosphatase. Advanced diagnostic techniques/gene assay were not done due to limitations in our resources. Based on clinical and radiographic findings, a provisional diagnosis of nonsyndromic oligodontia was made with a differential diagnosis of hypohidrotic variant of ectodermal dysplasia, as well as irreversible pulpitis of Tooth #11 secondary to trauma.

#### 2.2.4. Treatment

**Figure 9:**
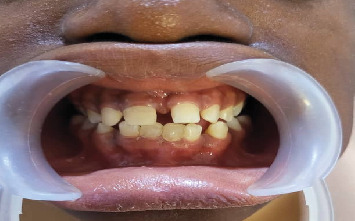
Clinical intraoral picture showing composite resin restoration of the peg-shaped crown of Tooth #12 and post and core restoration of the endodontically treated Tooth #11.

The treatment plan involved root canal treatment of the fractured Tooth #11 with post, core, and crown restoration; composite resin crown build-up of the peg-shaped Tooth #12; and aesthetic crown build-up of retained Teeth #71 and #81. After obtaining parental consent, root canal treatment on the fractured Tooth #11 commenced and was completed with its post, core, and crown restoration on their next visit. Also, the composite crown build-up was done on their second visit on the small-sized Teeth #71 and #81 and the peg-shaped Tooth #12 (Figure 9).

#### 2.2.5. Outcome

The patient and parents expressed satisfaction with functional and aesthetic outcomes.

## 3. Discussion

Oligodontia, as a form of tooth agenesis, has been described as a congenital or developmental absence of multiple permanent teeth [2, 12]. While some authors assert oligodontia to be the absence of six or more permanent teeth (excluding third molars), others refer to ‘hypodontia' as the absence of one to five permanent teeth, while also referring to ‘anodontia' as the absence of all permanent teeth [2, 11, 13].

Oligodontia has been extensively studied and termed a relatively rare condition with varying incidence reported from different geographic regions and ethnicities in the range of 0.08%–1.16% [5]. While a 0.19% prevalence was reported by Bergstrom et al. in a Swedish study [14], 0.1% was also reported in a Finnish population [15]. Also, Rølling et al. reported 0.16% of the Danish school children [2], while Steen et al. reported 0.08% in a Dutch population [16]. While there are extremely limited reported cases of oligodontia from Africa, the prevalence of oligodontia in a white population of North America, Australia, and Europe was reported to be 0.14% [17] and also 0.25% as reported by Feng in an Asian population of China [18]. Furthermore, the prevalence of oligodontia varies among different age groups. Studies done in Europe reported 0.16% oligodontia to occur in school children at 9–13 years of age [2], while 0.084% among 18-year-old adults [1]. The frequency of oligodontia was documented to affect females more often than males [19], with a gender ratio of 3:2 [20, 21]. The study done on the Danish school children reported female predilection for the anomaly [2], while another study by Goya et al. in Japan reported no significant difference between the occurrence in females and males [22].

The precise etiology for oligodontia is still not comprehensively unraveled. Several theories have been propagated to explain the etiology of oligodontia; most of which were focused on either environmental or genetic factors [23]. Certain environmental influences have long been recognized as risk factors for certain craniofacial anomalies like oligodontia, such as trauma involving the alveolar process disrupting tooth germ development, the contagious viral infection, ‘rubella' (German measles) during pregnancy, toxins, and radiotherapy or chemotherapy in early infancy [24–26]. Also, some studies reported that certain uterine conditions, such as maternal consumption of teratogenic substances like thalidomide during the first trimester of pregnancy, could contribute to the etiology of oligodontia or hypodontia. Studies reported that children with thalidomide embryopathy had a 7.7% higher tendency to manifest with hypodontia or oligodontia compared to normal children (0.4%) [24, 27]. However, it is interesting to note that one study reported that maternal health during pregnancy was found to be unrelated to the expression of hypodontia or oligodontia [28].

Tooth development is largely under genetic control, as also hypodontia or oligodontia [29]. Previously, studies on the etiologic factors of hypodontia or oligodontia focused on phenotypic presentations of families to find relevant genetic variants, but in recent studies, a good number of studies have focused on identifying the specific genes involved in regulating tooth development. Numerous genes are involved in tooth morphogenesis, including *MSX1*, *PAX9*, *EDA*, *SPRY2*, *SPRY4*, *TGFA*, *AXIN2*, *WNT10A*, *FGFR2*, *FGF3*, *FGF10*, and *BMP4* [30–32]. Of all these, *MSX1*, *PAX9*, *EDA*, and *AXIN2* genes are most frequently associated with nonsyndromic hypodontia or oligodontia [33–37]. All these genes have been confirmed to play vital roles in the signaling pathways and signal transduction cascades [30]. *PAX9* is a transcription factor expressed on the tooth mesenchyme during tooth development [36], and its mutation can halt tooth formation at the bud stage of tooth formation. Hence, heterozygous *PAX9* mutations in humans are linked to nonsyndromic tooth agenesis [38]. *MSX1* (Muscle Segment Homeobox 1) is expressed on the condensing ectomesenchyme of the tooth germ [39]. Mutations in the *MSX1* gene prematurely terminate tooth development. A study recently identified an *MSX1* frameshift mutation in a family with missing second premolars and mandibular central incisors [40]. *AXIN2* (Axis Inhibition Protein 2) regulates cell growth and cell proliferation and also negatively regulates the Wnt signaling pathway. This gene has been implicated in lower incisor agenesis [32, 41]. *EDA* (ectodysplasin A) encodes a protein (TNF-Ligand protein) involved in X-linked hypohidrotic ectodermal dysplasia, characterized by sparse hair, lack of sweat glands, and microdontia [23]. *EDA* and *EDA* receptor mutations have been reported to be associated with sporadic hypodontia or oligodontia [42] including missing maxillary lateral incisors [30]. Our cases demonstrated the phenotypic variability of oligodontia, from isolated tooth agenesis to complex presentations with systemic features. Case 1 represents classical isolated oligodontia with bilateral symmetrical tooth absence, consistent with autosomal dominant inheritance patterns associated with *PAX9* mutations. The absence of syndromic features and negative family history suggests either de novo mutations or incomplete penetrance. Case 2 presented a more complex phenotype with positive family history and mild systemic features. The presence of acanthosis nigricans, nail abnormalities, and hypohidrosis, while not fulfilling criteria for classic ectodermal dysplasia, suggests possible involvement of the *EDA* pathway. The asymmetrical tooth loss pattern and taurodont-like morphology were consistent with reports linking *MSX1* mutations to variable expressivity in oligodontia. However, the limited genetic testing availability in our setting precluded definitive molecular diagnosis, highlighting resource constraints in developing countries.

In this present study, Case 2 presented with peculiar dental features, including peg-shaped Tooth #12, taurodont-like Teeth #16 and #26, microdontia of teeth #71 and #81, and delayed exfoliation of deciduous Teeth #71, #81, #55, #65, #75, and #85 (Figure 8 and Table 1). This is consistent with the documented features of oligodontia [11, 15, 43]. However, Case 1 presented with generalized/multiple spacing on the anterior regions of the maxillary and mandibular jaws, which is also a classical dental feature of oligodontia (Figure 2 and Table 1). Other commonly encountered dental features include delayed teeth development and eruption, ectopic eruption, rotations of teeth adjacent to missing mandibular second premolars, alteration and reduction in tooth dimensions and morphology (e.g., conical or peg-like shape of canines), enamel hypoplasia, and shortening of roots [11, 15]. Microdontia, which seems to be the commonest dental feature of hypodontia or oligodontia, can affect one or more teeth and may be seen in either deciduous or permanent dentition [44]. Additionally, microdontia is genetically acquired and presents in its severest form in the condition of ectodermal dysplasia [44]. It is also a dental feature in patients who had radiation therapy or chemotherapy to the jaws in early childhood [45]. Delayed tooth development and eruption, a feature of hypodontia or oligodontia, is characterized by the absence of a permanent successor, thus delaying normal resorption of the roots of the deciduous teeth and preventing the timely exfoliation and retention of the deciduous teeth up to mid-adult years [43]. Ectopic eruption of the permanent teeth may arise due to the absence of the adjacent teeth, which usually serve to guide them during eruption [46].

Remarkable skeletal features evident in patients with hypodontia or oligodontia include reduction of mandibular plane angles, which is intricately associated with a reduced anterior facial height and lip protrusion [47]. Other features include reduced maxillary and mandibular anterior-posterior dimensions, a tendency to develop a Class III skeletal relationship [48] and increased freeway space, which in conjunction with reduced facial height, may present an altered facial appearance of overclosed jaws extraorally [44].

Oligodontia and other forms of severe tooth agenesis may result in significant negative psychosocial and functional effects on the individuals affected. Both cases presented in this study demonstrated significant psychosocial impact, particularly regarding self-esteem and social interaction. This aligns with studies by Hobkirk et al. [49] and Laing et al. [50] reporting correlations between oligodontia severity and quality of life measures. Affected subjects with retained deciduous teeth at the time of presentation had fewer complaints because their unexfoliated deciduous teeth masked the problem.

Treatment of an oligodontia patient requires a multidisciplinary approach that integrates insights from prosthodontics, orthodontics, oral and maxillofacial surgery, genetics, pediatric dentistry, and pediatric medicine [11]. Treatment options may vary depending on the severity of the condition, age, patient's cardinal complaints, condition of retained deciduous teeth, occlusion, saddle spaces, alveolar ridge geometry, condition of supporting tissues, and the doctor's perceived need for care [51]. Usually, because of its potential complications, such as speech difficulties and masticatory problems, prompt treatment is advised. Prosthodontic intervention is the mainstay, especially in the early presentation of anxious patients with aesthetic and functional concerns [51]. Sometimes, prerestorative orthodontic therapy may be required to move displaced teeth to a favorable position also be instituted. Our management strategies reflect adaptive approaches necessitated by resource limitations while maintaining therapeutic efficacy. The prosthetic rehabilitation in Case 1 successfully addressed functional and aesthetic concerns, consistent with literature supporting early intervention benefits. The conservative approach in Case 2, utilizing composite restorations and endodontic therapy, demonstrated effective outcomes with limited resources.

Though international treatment protocols emphasize multidisciplinary care, our resource-constrained setting encouraged prioritizing immediate functional needs, while planning staged interventions proved more practical. Our outcomes support the effectiveness of simplified approaches when comprehensive care is unavailable. The improvement in confidence following treatment in both cases emphasizes the importance of early intervention beyond purely functional considerations.

The absence of a genetic screening protocol (‘Tooth agenesis panel'—NGS) represents a significant limitation in our study, preventing definitive etiological classification. Additionally, long-term follow-up data would strengthen outcome assessments. Future research should focus on establishing genetic screening protocols feasible for resource-limited settings and developing treatment guidelines adapted to local constraints.

The establishment of regional oligodontia registries could facilitate a better understanding of disease patterns in African populations and guide resource allocation for specialized care. Collaboration with international organizations could enhance access to advanced diagnostic and therapeutic modalities.

To enhance accuracy and transparency, this case report was presented in adherence to the CAse REport (CARE) guideline [52].

## 4. Conclusion

Oligodontia presents significant challenges in resource-limited settings, requiring adaptive management approaches while maintaining therapeutic effectiveness. Our cases demonstrate successful outcomes using available resources, emphasizing the importance of early diagnosis and patient-centered care. The phenotypic variability observed highlights the need for comprehensive clinical assessment even when advanced genetic testing is unavailable.

Future efforts should focus on developing sustainable healthcare models that integrate specialized oligodontia care into existing health systems. International collaboration and resource sharing could significantly improve access to optimal care for affected individuals in developing countries.

The successful management of these cases demonstrates that effective treatment is achievable within resource constraints, providing hope for improved quality of life for oligodontia patients in similar settings worldwide.

## Figures and Tables

**Table 1:** Comparison of the cases.

**Parameter**	**Case 1**	**Case 2**
Demographics	10-year-old female	10-year-old female
Missing teeth	12 permanent teeth	10 permanent teeth
Pattern	Bilateral symmetrical	Asymmetrical
Family history	Negative	Positive (sister affected)
Systemic features	Absent	Present (acanthosis nigricans, hypohidrosis)
Dental anomalies	Delayed eruption, generalized spacing	Microdontia, taurodontia, peg-shaped tooth #12
Treatment approach	Prosthetic replacement	Conservative restoration
Primary concern	Aesthetics, function	Pain, aesthetics

## Data Availability

The data supporting the findings of this study are available within the article. For any inquiries regarding data access or if you require additional information, please get in touch with the corresponding author through bernard.ogbozor@unn.edu.ng.

## References

[1] Nordgarden H., Jensen J. L., Storhaug K. (2002). Reported Prevalence Of Congenitally Missing Teeth in Two Norwegian Counties. *Community Dental Health*.

[2] Rølling S., Poulsen S. (2001). Oligodontia in Danish School Children. *Acta Odontologica Scandinavica*.

[3] Al-Ani A. H. (2016). *Genetic and Environmental Factors Associated With Hypodontia*.

[4] Lee K. E., Ko J., Shin T. J., Hyun H. K., Lee S. H., Kim J. W. (2014). Oligodontia and Curly Hair Occur With Ectodysplasin-A Mutations. *Journal of Dental Research*.

[5] Singer S. L., Henry P. J., Lander I. D. (2010). A Treatment Planning Classification for Oligodontia. *International Journal of Prosthodontics*.

[6] Noor A., Windpassinger C., Vitcu I. (2009). Oligodontia Is Caused by Mutation in LTBP3, the Gene Encoding Latent TGF-*β* Binding Protein 3. *American Journal of Human Genetics*.

[7] Mostowska A., Biedziak B., Trzeciak W. H. (2006). A Novel c. 581C> T Transition Localized in a Highly Conserved Homeobox Sequence of MSX1: Is It Responsible for Oligodontia?. *Journal of Applied Genetics*.

[8] Hosur M. B., Puranik R. S., Vanaki S. S. (2011). Oligodontia: A Case Report and Review of Literature. *World Journal of Dentistry*.

[9] Kotsiomiti E., Kassa D., Kapari D. (2007). Oligodontia and Associated Characteristics: Assessment in View of Prosthodontic Rehabilitation. *European Journal of Prosthodontics and Restorative Dentistry*.

[10] Scarfe W. C., Farman A. G., Sukovic P. (2006). Clinical Applications of Cone-Beam Computed Tomography in Dental Practice. *Journal-Canadian Dental Association*.

[11] Worsaae N., Jensen B. N., Holm B., Holsko J. (2007). Treatment of Severe Hypodontia–Oligodontia—An Interdisciplinary Concept. *International Journal of Oral and Maxillofacial Surgery*.

[12] Abu-Hussein M., Watted N., Zere E. (2015). Nonsyndromic Oligodontia in Permanent Dentition: Three Rare Cases. *IOSR Journal of Dental and Medical Sciences (IOSR-JDMS)*.

[13] Durstberger G., Čelar A., Watzek G. (1999). Implant-Surgical and Prosthetic Rehabilitation of Patients With Multiple Dental Aplasia: A Clinical Report. *International Journal of Oral & Maxillofacial Implants*.

[14] Bergstrom K. (1977). An Orthopantomographic Study of Hypodontia, Supernumeraries and Other Anomalies in School Children Between the Ages of 8-9 Years. An Epidemiological Study. *Swedish Dental Journal*.

[15] Nieminen P. (2009). Genetic Basis of Tooth Agenesis. *Journal of Experimental Zoology Part B: Molecular and Developmental Evolution*.

[16] Steen W. H., Bosman F. (1992). Distribution of Missing Teeth and Tooth Morphology in Patients With Oligodontia. *ASDC Journal of Dentistry for Children*.

[17] Creton M. A., Cune M. S., Willem Verhoeven J., Meijer G. J. (2007). Patterns of Missing Teeth in a Population of Oligodontia Patients. *International Journal of Prosthodontics*.

[18] Feng H.-L. (2011). Prosthodontic Treatment of Congenital Tooth Agenesis I. The Classicfication, Prevalence and Etiology of Congenital Tooth Agenesis. *Zhonghua kou qiang yi xue za zhi= Zhonghua kouqiang yixue zazhi= Chinese Journal of Stomatology*.

[19] Polder B. J., van’t Hof M. A., van der Linden F. P. G. M., Kuijpers-Jagtman A. M. (2004). A Meta-Analysis of the Prevalence of Dental Agenesis of Permanent Teeth. *Community Dentistry and Oral Epidemiology*.

[20] Bural C., Oztas E., Ozturk S., Bayraktar G. (2012). Multidisciplinary Treatment of Non-Syndromic Oligodontia. *European Journal of Dentistry*.

[21] Dermaut L. R., Goeffers K. R., De Smit A. A. (1986). Prevalence of Tooth Agenesis Correlated With Jaw Relationship and Dental Crowding. *American Journal of Orthodontics and Dentofacial Orthopedics*.

[22] Goya H. A., Tanaka S., Maeda T., Akimoto Y. (2008). An Orthopantomographic Study of Hypodontia in Permanent Teeth of Japanese Pediatric Patients. *Journal of Oral Science*.

[23] Galluccio G., Castellano M., La Monaca C. (2012). Genetic Basis of Non-Syndromic Anomalies of Human Tooth Number. *Archives of Oral Biology*.

[24] Brook A. H. (2009). Multilevel Complex Interactions Between Genetic, Epigenetic and Environmental Factors in the Aetiology of Anomalies of Dental Development. *Archives of Oral Biology*.

[25] Cameron J., Sampson W. J. (1996). Hypodontia of the Permanent Dentition. Case Reports. *Australian Dental Journal*.

[26] Näsman M., Forsberg C.-M., Dahllöf G. (1997). Long-Term Dental Development in Children After Treatment for Malignant Disease. *European Journal of Orthodontics*.

[27] Gilbert-Barness E. (2010). Teratogenic Causes of Malformations. *Annals of Clinical & Laboratory Science*.

[28] Boruchov M. J., Green L. J. (1971). Hypodontia in Human Twins and Families. *American Journal of Orthodontics*.

[29] Jernvall J., Thesleff I. (2000). Reiterative Signaling and Patterning During Mammalian Tooth Morphogenesis. *Mechanisms of Development*.

[30] Alves-Ferreira M., Pinho T., Sousa A., Sequeiros J., Lemos C., Alonso I. (2014). Identification of Genetic Risk Factors for Maxillary Lateral Incisor Agenesis. *Journal of Dental Research*.

[31] Kapadia H., Mues G. D. S. R., D'souza R. (2007). Genes Affecting Tooth Morphogenesis. *Orthodontics & Craniofacial Research*.

[32] Küchler E. C., Lips A., Tannure P. N. (2013). Tooth Agenesis Association With Self-Reported Family History of Cancer. *Journal of Dental Research*.

[33] Mues G., Tardivel A., Willen L. (2010). Functional Analysis of Ectodysplasin-A Mutations Causing Selective Tooth Agenesis. *European Journal of Human Genetics*.

[34] Hansen L., Kreiborg S., Jarlov H., Niebuhr E., Eiberg H. (2007). A Novel Nonsense Mutation inPAX9is Associated With Marked Variability in Number of Missing Teeth. *European Journal of Oral Sciences*.

[35] Nikopensius T., Annilo T., Jagomägi T. (2013). Non-Syndromic Tooth Agenesis Associated With a Nonsense Mutation in Ectodysplasin-A (EDA). *Journal of Dental Research*.

[36] Mitsui S. N., Yasue A., Masuda K. (2014). Novel PAX9 Mutations Cause Non-Syndromic Tooth Agenesis. *Journal of Dental Research*.

[37] Das P., Stockton D. W., Bauer C. (2002). Haploinsufficiency of PAX9 Is Associated With Autosomal Dominant Hypodontia. *Human Genetics*.

[38] Cobourne M. T., Sharpe P. T. (2013). Diseases of the Tooth: The Genetic and Molecular Basis of Inherited Anomalies Affecting the Dentition. *Wiley Interdisciplinary Reviews: Developmental Biology*.

[39] Mackenzie A., Ferguson M. W. J., Sharpe P. T. (1992). Expression Patterns of the Homeobox Gene, Hox-8, in the Mouse Embryo Suggest a Role in Specifying Tooth Initiation and Shape. *Development*.

[40] Kim J.-Y., Cha Y. G., Cho S. W. (2006). Inhibition of Apoptosis in Early Tooth Development Alters Tooth Shape and Size. *Journal of Dental Research*.

[41] Callahan N., Modesto A., Meira R., Seymen F., Patir A., Vieira A. R. (2009). Axis Inhibition Protein 2 (AXIN2) Polymorphisms and Tooth Agenesis. *Archives of Oral Biology*.

[42] Bergendal B., Klar J., Stecksén-Blicks C., Norderyd J., Dahl N. (2011). Isolated Oligodontia Associated With Mutations in EDARADD, AXIN2, MSX1, and PAX9 Genes. *American Journal of Medical Genetics Part A*.

[43] Haselden K., Hobkirk J. A., Goodman J. K., Jones S. P., Hemmings K. W. (2001). Root Resorption in Retained Deciduous Canine and Molar Teeth Without Permanent Successors in Patients With Severe Hypodontia. *International Journal of Paediatric Dentistry*.

[44] Hobkirk J., Gill D. S., Jones S. P. (2010). Part 1: Background. *Hypodontia: A Team Approach to Management*.

[45] Oğuz A., Çetiner S., Karadeniz C., Alpaslan G., Alpaslan C., Pinarli G. (2004). Long-Term Effects of Chemotherapy on Orodental Structures in Children With Non-Hodgkin’s Lymphoma. *European Journal of Oral Sciences*.

[46] Al-Ani A. H., Antoun J. S., Thomson W. M., Merriman T. R., Farella M. (2017). Hypodontia: An Update on Its Etiology, Classification, and Clinical Management. *BioMed Research International*.

[47] Chung L.-K. L., Hobson R. S., Nunn J. H., Gordon P. H., Carter N. E. (2000). An Analysis of the Skeletal Relationships in a Group of Young People With Hypodontia. *Journal of Orthodontics*.

[48] Øgaard B., Krogstad O. (1995). Craniofacial Structure and Soft Tissue Profile in Patients With Severe Hypodontia. *American Journal of Orthodontics and Dentofacial Orthopedics*.

[49] Hobkirk J. A., Goodman J. R., Jones S. P. (1994). Presenting Complaints and Findings in a Group of Patients Attending a Hypodontia Clinic. *British Dental Journal*.

[50] Laing E., Cunningham S. J., Jones S., Moles D., Gill D. (2010). Psychosocial Impact of Hypodontia in Children. *American Journal of Orthodontics and Dentofacial Orthopedics*.

[51] Finnema K. J., Raghoebar G. M., Meijer H. J., Vissink A. (2005). Oral Rehabilitation With Dental Implants in Oligodontia Patients. *International Journal of Prosthodontics*.

[52] (2013). Case Reports – The CARE Statement. *Evidence-Based Dentistry*.

